# Modeling Pharmacokinetic Profiles for Assessment of Anti-Cancer Drug on a Microfluidic System

**DOI:** 10.3390/mi11060551

**Published:** 2020-05-29

**Authors:** Yaqiong Guo, Pengwei Deng, Wenwen Chen, Zhongyu Li

**Affiliations:** 1 Division of Biotechnology, CAS Key Laboratory of Separation Sciences for Analytical Chemistry, Dalian Institute of Chemical Physics, Chinese Academy of Sciences, Dalian 116023, China; guoyaqiong@dicp.ac.cn (Y.G.); dengpengwei@dicp.ac.cn (P.D.); chenwenwen@dicp.ac.cn (W.C.); 2 University of Chinese Academy of Sciences, Beijing 100190, China; 3 College of Life Science, Dalian Minzu University, Dalian 116600, China

**Keywords:** microfluidics, intestine, drug, pharmacokinetic (PK) profile

## Abstract

The pharmacokinetic (PK) properties of drug, which include drug absorption and excretion, play an important role in determining the in vivo pharmaceutical activity. However, current in vitro systems that model PK profiles are often limited by the in vivo-like concentration profile of a drug. Herein, we present a perfused and multi-layered microfluidic chip system to model the PK profile of anti-cancer drug 5-FU in vitro. The chip device contains two layers of culture channels sandwiched by a porous membrane, which allows for drug exposure and diffusion between the two channels. The integration of upper intestine cells (Caco-2) and bottom targeted cells within the device enables the generation of loading and clearance portions of a PK curve under peristaltic flow. Fluorescein as a test molecule was initially used to generate a concentration-time curve, investigating the effects of parameters of flow rate, administration time, and initial concentration on dynamic drug concentration profiles. Furthermore, anti-cancer drug 5-FU was performed to assess its pharmaceutical activity on target cells (human lung adenocarcinoma cells or human pulmonary alveolar epithelial cells) using different drug administration regimens. A dynamic, in vivo-like 5-FU exposure refers to PK profile regimen, led to generate a lower drug concentration (dynamically fluctuate from 0 to 1 μg/mL affected by absorption) compared to the constant exposure. Moreover, the PK profile regimen alleviates the drug-induced cytotoxicity on target cells. These results demonstrate the feasibility of determining the PK profiles using this microfluidic system with in vivo-like drug administration regimens. This established system may provide a powerful platform for the prediction of drug safety and effectiveness in the pharmaceutical research.

## 1. Introduction

Pharmacokinetic (PK) properties of a drug play a crucial role in determining the pharmacological activity in human subjects [1,2,3]. It could capture the dynamic concentration profiles of drugs and provide a quantitative description of the relationship between drug exposure and drug response in pharmaceutical research. Currently, a typical protocol for studying PK profile is predominantly based on animal tests [4,5,6]; however, animal experiments are associated with various defects, such as ethical issues, species differences, and high cost. Additionally, the monolayer cell culture has been widely performed as an alternative to animal experiments [7]. Cell-based assays always soak the target cells in the drug-containing medium stably, which is quite different from the dynamic changes of drug concentration in the body [8,9]. Thereby, cells model fails to recapitulate the PK profile and biologically relevant response which cells experience in physiological systems. It is highly desired to establish practical and reliable drug testing models with a physiologically-based PK profile. 

Recent advances in microfluidic techniques have led to the development of in vitro tissues models with improved tissues morphology and function. And it shows significant promise for drug assessment compared to conventional research [10,11,12,13,14]. In PK studies, the intestine is important as it is closely related to the bioavailability and bioactivity of ingested substances [15,16]. Microfluidic systems could generate a physiologic microenvironment for a variety of tissues, including the intestine, and greatly shorten the construction period of the in vitro human models [17,18,19,20]. Furthermore, various microfluidic systems have been developed to recreate physiological tissue organization or inter-tissue interactions, which have been proposed for the prediction of PKs [21,22]. Although a lot of progress has been made for drug testing using microfluidic systems [23,24,25], few attempts have been made to model concentration-time curve of drug, which is essential for PK profile drug testing [26].

In this study, we designed and developed a multi-layered perfused microfluidic system that allows us to capture the concentration-time curve of drugs in vitro. The microfluidic system consists of compartmentalized chambers facilitating the co-culture of different cell types. Peristaltic flow through this system generates low level of shear stress to improve intestinal barrier function and enhance cell layer integrity. The dynamic flow protocol enables both loading and clearance portions of drug exposure to be generated. Generation of an optimized concentration-time curve by adjusting flow parameter (flow rate in the upper channel, flow rate in the bottom channel, concentration of fluorescein, and administration time) was initially performed with fluorescein as a test molecule. An approved antineoplastic 5-FU was utilized as the test drug for generated concentration-time curve with the PK profile on the chip. Moreover, the effects of 5-FU on the target cells were assessed, using different drug administration regimens.

## 2. Materials and Methods

### 2.1. Materials

Materials included polydimethylsiloxane (PDMS, Dow Corning Corp., Auburn, AL, USA), SU-8 3035 photoresist (MicroChem Corp., Westborough, MI, USA), porous membrane (0.4 μm, Whatman Corp., Buckinghamshire, UK), 96-well plates(Guangzhou Jet Bio-filtration Co., Ltd., Guangzhou, China), fetal bovine serum (FBS, Gibco, Grand Island, NE, USA), Dulbecco’s modified eagle medium (DMEM, Gibco, Grand Island, NE, USA), trypsin-EDTA (Gibco, Grand Island, NE, USA), cell counting kit-8 (CCK-8, Dojindo, Kumamoto, Japan), Live/Dead Kit (BD), and 5-FU (Casmart Mall, Beijing, China). 

### 2.2. Device Design and Fabrication

**Figure 1:**
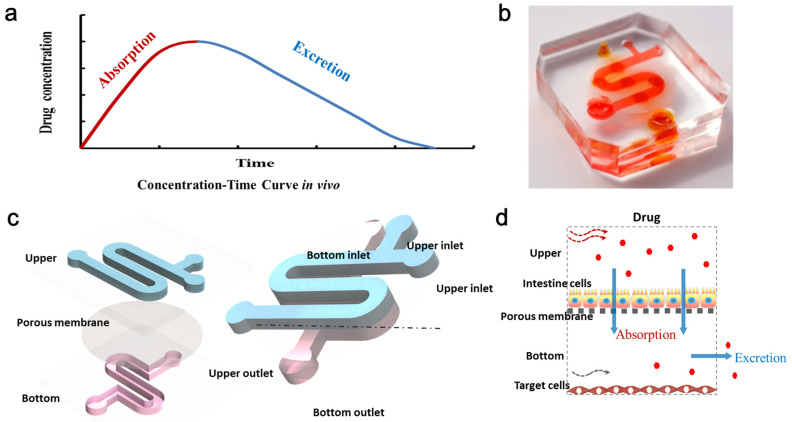
Schematic diagram of the multilayered microfluidic chip for modeling pharmacokinetic (PK) profiles of drug administration. (**a**) The concentration-time curve in vivo. (**b**) The actual image of the multilayered chip. (**c**) The configuration of the chip device. The device is composed of three layers, including the S-shaped upper channel loading with intestine cells, porous membrane, and bottom channel loading with targeted cells. (**d**) Schematic view of the PK process of absorption and excretion on cells with drug exposure in the device.

The microfluidic system was fabricated, as previously reported, using soft lithography and micromolding [27]. The masks were designed by AutoCAD (Autodesk) and printed on plastic film with 4000 dpi resolution. Firstly, to prepare the template, SU-8 photoresist was spin-coated onto two clean glass wafers and then selectively cured under an ultraviolet light source by using different masks. Afterwards, the mixture of PDMS monomer and curing agent at the ratio of 6:1 by mass was used for generating two layers’ PDMS replicas. Finally, the two PDMS replicas were sealed together with the middle porous membrane. As shown in Figure 1, the microfluidic chip consists of two PDMS layers and a porous membrane (0.4 μm pore size). The upper layer of the chip had one S-shaped channel with 100 μm in height, 1 mm in width and 40 mm in length, and the bottom layer had one analogous channel that was 300 μm in height. The bottom channel was designed for sampling in and out with one inlet and one outlet. For the convenience of drug treatment, the upper channel was designed with two inlets and one outlet. 

### 2.3. Computational Simulation of Flow

The fluidic flow was simulated with COMSOL Multiphysics 5.4, as previously reported. Automatic remeshing performed a time-related study according to the fluid velocity field in the solution for analyzing the flow rate and shear stress.

### 2.4. Cell Culture

Human colorectal cells (Caco-2, Cell Bank of the Chinese Academy of Sciences, Shanghai, China), human lung adenocarcinoma cells (A549, Cell Bank of the Chinese Academy of Sciences, Shanghai, China) and human alveolar epithelial cells (HPAEpiC, Sciencell Corporation, Shanghai, China) were cultured in DMEM medium supplemented in the cell incubator. The chambers of the chip were coated with 8 μg/mL Collagen I for 12 h, contributing to cell seeding and culture. At 80% confluence, Caco-2 cells were firstly injected into the upper channel at a density of 10^5^ cells/mL. After trypsinization in the dishes, Caco-2 cells were seeded into the chamber overnight until cell attachment. Then, perfusion medium by the micro-pump was initiated through the upper channel at fluid shear stress 0.02 dyne/cm^2^ for 4 days, until cells grew to form intact monolayer. The fluid shear stress is similar to the physiological flow rate present in gut [17,19,28]. According to contractions of 9–11/min in vivo [29], the peristaltic flow is forward 4.5 s and backward 1.5 s as a continuous loop, with the velocity in the forward 60 μL/h. A549 and HPAEpiC cells were centrifuged and resuspended to the density of 10^6^ cells/mL. Then, cells were firstly seeded into the lower cell culture channel and cultured. After that, the microchip was placed in the cell culture incubator for 24 h, followed by drug stimulation.

### 2.5. Molecular Permeability Assay of Intestine Barrier

The Caco-2 monolayer of microfluidic chip was maintained in the different fluid conditions for 4 days. Then, the Fluorescein Isothiocyanate-labeled dextran (FD20, 20 kDa) solution (1 mg/mL) was perfused through the upper channel, and fluorescence intensity of the sample aliquots collected every hour from the bottom channel were measured at 490 nm /520 nm. The apparent permeability coefficient (Papp) was calculated according to Papp (cm/s) = (dQ/dt)(1/AC_0_). 

### 2.6. Immunohistochemistry

By staining the tight junction protein ZO-1 using a confocal immunofluorescence microscope, the establishment of tight junction between cells at the top was evaluated. After being drug stimulated, Caco-2 cells were fixed with 4% paraformaldehyde for 15 min. The Caco-2 cells used 0.2% Triton-X100 for permeabilizing before incubation with ZO-1 antibody for 12 h at 4 °C. Then, samples were incubated with fluorescently-labeled antibody for 1 h, followed by counterstaining with 4’,6-diamidino-2-phenylindole (DAPI) for 15 min. After washing with PBS, the microscopy was performed using a Leica laser confocal microscope.

### 2.7. Analysis of Fluorescein

Fluorescein solution was pumped through the chip in various ways (flow rate in the upper channel 30, 60, 120 μL/h; flow rate in the bottom channel 0.5, 1, 2 μL/h; concentration of fluorescein 10, 20, 40 μg/mL; loading time 1, 2, 4 h). The medium containing fluorescein were as sampled hourly from the bottom layer. The solution was collected into a 96-well plate and then the absorbance at 490 nm /520 nm was measured by a microplate reader.

### 2.8. Sample Preparation and MS Detection

The 5-FU absorbed in microfluidic system was detected. The medium in the bottom channel were collected, respectively, at different points over 48 h. One microliter of medium containing 5-FU absorbed was added to 0.05 μL methanoic acid and 50 μL ethyl acetate. After centrifuging for 8 min at 3000 rpm, 40 μL of the top layer was transferred into a new tube. Once dried and re-dissolved in 40 μL of the mobile phase, it consisted of acetonitrile and water (80:20, *v*/*v*). The sample was injected into the liquid chromatography–tandem mass spectrometry (LC-MS/MS) for analysis.

### 2.9. Cytotoxicity Assessment

In the PK profile regimen (Pk group), we mimicked drug absorption and clearance profiles for a human orally twice daily by starting cell exposure of 5-FU (20 μg/mL) for 2 h and stopping exposure for 10 h over a 12 h period. In a constant profile regimen (Con group), we continuously infused 5-FU (20 μg/mL) for 48 h.

The medium containing 5-FU (20 μg/mL) was pumped into the Caco-2 cell culture chambers using two regimens for assessing drug activity and toxicity on the chip. In the intestine, 5-FU was absorbed and then flowed into the lower cell chambers in which target cells were stimulated by absorbed 5-FU. After 48 h of drug stimulation, cells were stained using the Live/Dead Kit, and quantitative data of cell vitality were tested by CCK-8 assay. CCK-8 reagent was added into the cells culture channel. The solution was collected into a 96-well plate and then the absorbance at 450 nm was measured by a microplate reader after incubation for 2 h. 

### 2.10. Statistical Analysis

In this study, all image analysis was performed using ImageJ image software, all data were expressed as means±standard error, and all experiments were repeated at least three times. Comparisons of two groups were performed by using the two-sample *t*-test. *p* < 0.05 was considered statistically significant.

## 3. Results and Discussion

### 3.1. The Design of Microfluidic Device

In this work, a three-layer microfluidic chip device was designed and fabricated. The chip was micro-fabricated with clear, flexible, polydimethylsiloxane (PDMS), as described previously [30]. The chip consisted of an upper channel for the culture of intestine cells and a bottom layer with a corresponding micro-channel for the culture of target cells, including human lung adenocarcinoma cells (A549 cell line) and human alveolar epithelial cells (HPAEpiC cell line). Porous membrane enabled the transportation of drug to target cells in the bottom channel after absorption by intestine cells in the upper channel. The multi-layered design of the chip can be used to simulate the processes of absorption and excretion, holding great potential for the study of different drug administration regimens and assessment of drug absorption-dependent toxicity on target cells.

In humans, most drugs are absorbed primarily in the intestine. Therefore, intestine cell line Caco-2 was seeded into the upper channel for the study of intestinal drug absorption. To investigate the activity of anti-cancer drug 5-FU on chip under different administration conditions, the target cells were seeded into the bottom channel. 5-FU was added into the top chamber, and the drug activity on target cells could be examined.

**Figure 2:**
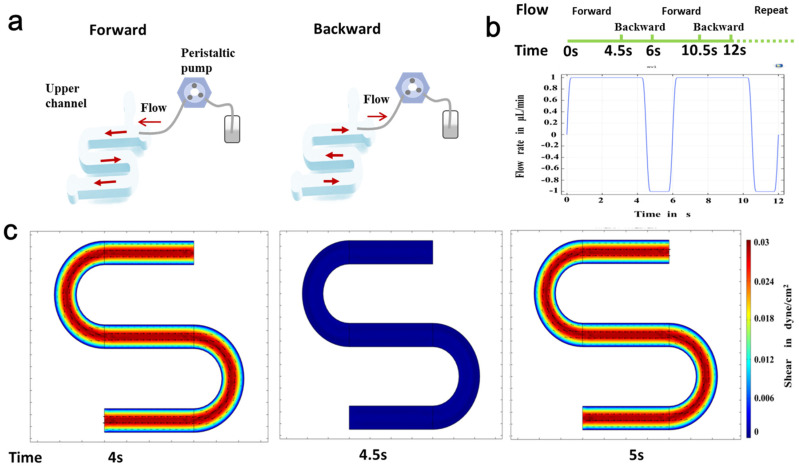
Mathematical simulation of the peristaltic flow in intestine on chip. (**a**) Schematic drawing of peristaltic flow in the upper channel of the chip device. (**b**) The changes of flow velocity as the periodical changes of flow direction. (**c**) The numerical simulation of shear stress in the upper channel by computational fluid dynamics analysis.

To mimic the near-physiological dynamic microenvironment, peristaltic flow was applied to the intestine monolayer on the top channel. Mathematical simulation of fluid flow was conducted to determine the range of shear stress levels. Figure 2b shows the flow velocity changes periodically as the transformation of flow direction. It is known that fluid flow is beneficial for intestinal cell growth and survival. As shown in the COMSOL-based computational fluid dynamics (CFD) simulation data (Figure 2c), the average shear stress in the channel was approximately 0.02~0.03 dyne/cm^2^, which is similar to the fluid flow in the intestine in vivo. Zero shear occurs at the moment when the fluid flow changes direction, and the hold time is extremely short.

### 3.2. Evaluation of Intestinal Cell Differentiation on Chip

Previous studies have highlighted the importance of dynamic flow to enhance differentiated intestinal barrier functions in comparison to the sustained condition [17,18,19]. However, the peristaltic flow, not the sustained flow, is the essential physiological transport mechanism in most of the gastrointestinal tract. Hence, to improve the physiological relevance of this microsystem, Caco-2 cells were grown under peristaltic flow as *in vivo*.

**Figure 3:**
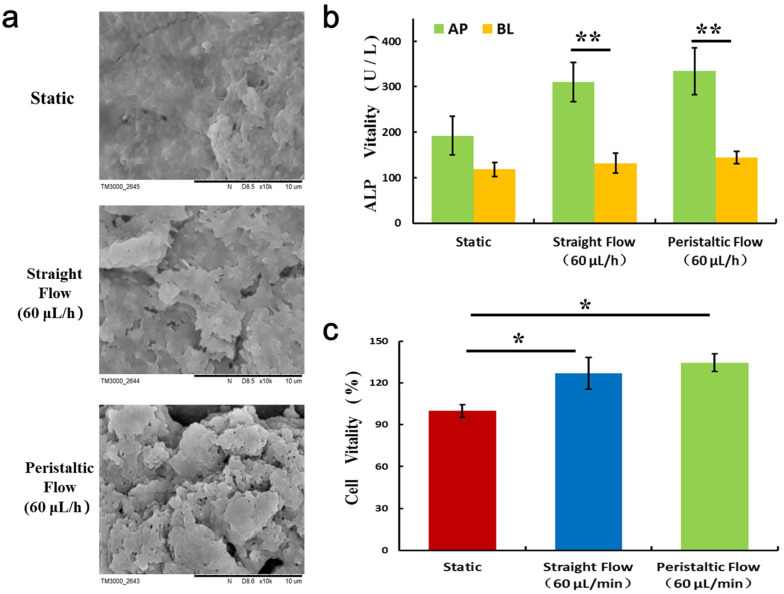
Characterization of intestinal differentiation of the Caco-2 monolayer under different culture conditions. (**a**) Scanning electron microscope (SEM) images of the morphology of Caco-2 on chip under static, straight flow, and peristaltic flow conditions. (**b**) The ALP vitality of intestinal cells under distinct cultures were assessed on apical (AP) and basolateral (BL) sides, respectively. Results were obtained from triplicate independent experiments (N = 3). Data are shown as mean ± SD. ** *p* < 0.01. (**c**) Quantified analysis of the Caco-2 cell viability by cell counting kit-8 (CCK-8) kit. N = 3, * *p* < 0.05.

Firstly, we examined cell morphology and function under peristaltic flow compared to sustained flow and static state. Electron microscopy analysis revealed that epithelial cells grown under static condition were flattened (Figure 3a). Moreover, cells grown in the dynamic flow at a rate of 0.02 dyne/cm^2^ shear stress were columnar. Thus, dynamic flow and shear stress could accelerate cell differentiation with the higher polarization after culture for 4 days, evidenced by the formation of characteristic intestinal villi of Caco-2 cells, as displayed in previous reports [17,18]. To further determine the differentiation of intestinal epithelial cells, we examined the alkaline phosphatase (ALP) activity of Caco-2 cells in different conditions. ALP is commonly used as a marker for intestinal epithelial cell differentiation as a brush border enzyme. The results showed that cell cultured with peristaltic flow in the microfluidic device got 3-fold higher ALP activity when compared to the cells cultured in static state.

### 3.3. Reconstitution of Intestinal Barrier Functions on Chip

**Figure 4:**
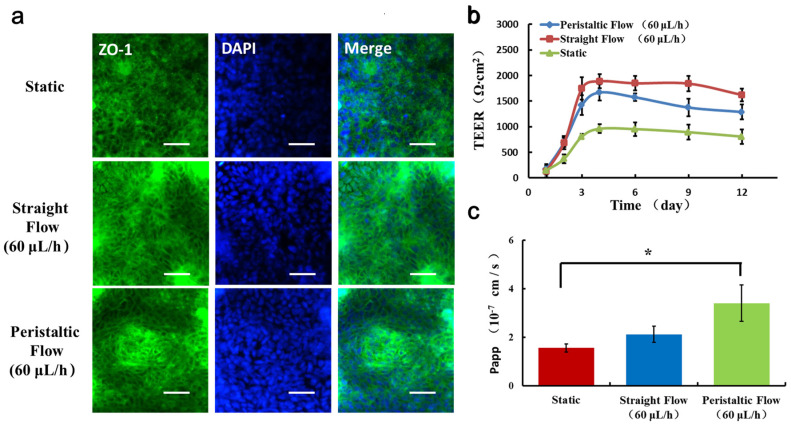
Evaluation of intestinal barrier functions under different flow conditions. (**a**) Immunofluorescent images show the expression of tight junction protein, ZO-1 (green), in the Caco-2 monolayer. Bars: 50 μm. (**b**) The barrier integrity of the Caco-2 monolayer was quantified by TEER. (**c**) The apparent permeability coefficient (Papp) was measured by quantifying FD20 transport across the Caco-2 monolayer under different conditions. N = 3, * *p* < 0.05.

To further assess the functional features of the intestinal monolayer on the chip, the structural and functional integrity of the intestinal barrier were examined (Figure 4). Caco-2 cells were observed to form confluent polygonal epithelial monolayers with well-developed tight junctions under dynamic flow conditions. The Caco-2 cells at confluence were well delimited by junctions and organized in a characteristic geometry, indicating the integrated tight junction assembly on chip. The trans-endothelial electrical resistance (TEER) value of the Caco-2 monolayer on chip was measured as previously reported [31]. The baseline resistance value of control sample (no cells) was measured and subtracted from that of the Caco2 monolayer samples under various conditions. These studies revealed that the TEER value of cells grown under dynamic flow conditions increased over the first 4–5 days and then maintained similarly high levels. Cells on the chip with straight flow and peristaltic flow displayed peak TEER levels higher than those of cells in static condition, reaching a value of 2000 Ω·cm^2^.

We also measured the Papp of the Caco-2 monolayer, which characterizes the barrier function. We found that the peristaltic flow induced higher permeability. These results are consistent with published studies that Caco-2 cell monolayers in static cultures display lower permeability values [32]. This low level of permeability could result from the presence of a thick unstirred fluid layer in the static culture, which might limit diffusion. The peristaltic flow would increase paracellular permeability by producing fluid shear stress that decreases the thickness of the unstirred diffusion layer. Obviously, these data suggest that the intestinal barrier was formed in our chip over the 4 days with dynamic flow. In previous studies, application of dynamic fluid flow could accelerates the intestinal epithelial cell differentiation [30]. The peristaltic flow perfusion culture utilized is essential for providing cells with appropriated shear stress.

### 3.4. Simulation of Absorption and Excretion Portions of Drug Using Fluorescein

**Figure 5:**
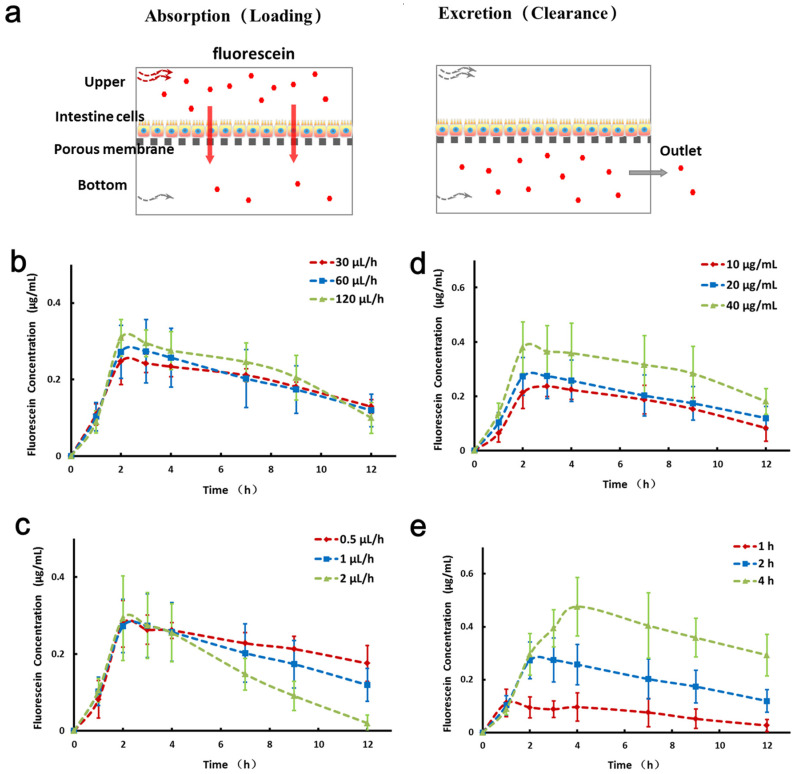
Simulation of dynamic drug absorption and excretion profiles using fluorescein on a microfluidic system. (**a**) Schematic drawing for the administration of fluorescein into the upper channel of device. The control parameters include flow rate, fluorescein concentration, and administration time. (**b**) Quantification of fluorescein concentration diffused over the bottom channel by varying the flow rate (30, 60, 120 μL/h) in the upper channel at different time points. The fluorescein concentration (20 μg/mL), administration time (2 h), and flow rate in the bottom channel (1 μL/h) are constant. (**c**) Quantified analysis of fluorescein concentration with different flow rates (0.5, 1, 2 μL/h) in the bottom channel. The administration time (2 h), fluorescein concentration (20 μg/mL), and flow rate in the upper channel (60 μL/h) are constant. (**d**) Varying the fluorescein concentrations (10, 20, 40 μg/mL) with fixed administration time (2 h) and flow rates in the upper channel (60 μL/h) and bottom channel (1 μL/h). (**e**) Varying administration time (1, 2, 4 h) of fluorescein administration with fixed concentration and flow rates.

The flow parameters in the microfluidic system were measured by injecting fluorescein. Parameters affecting the concentration-time curve including flow rate in the upper channel, flow rate in the bottom channel, concentration of fluorescein, and administration time were shown in Figure 5. Fluorescein was used as a test molecule for investigating all of the parameters mentioned above. The elimination profile was determined by delivery of fluorescein-free medium through the channel. The effect of flow parameters on the concentration-time curve was determined by maintaining the varying flow rate in the upper channel (30, 60, 120 μL/h), varying flow rate in the bottom channel (0.5, 1, 2 μL/h), varying concentration of fluorescein (10, 20, 40 μg/mL), and varying administration time (1, 2, 4 h).

The result showed that the varying flow rate in the upper channel has little impact on the PK profiles with fixed concentration of fluorescein, administration time, flow rate in the bottom channel. In contrast, a higher flow rate in the bottom channel (e.g., 2 μL/h) yields a reduced fluorescein concentration faster than that with lower flow in the same equivalent administration time, fluorescein concentration, and flow rate in the upper channel. Flow rate in the bottom channel directly affects the elimination, as well as the higher flow rate in the bottom channel with the faster elimination rate. The fluorescein at a higher concentration (e.g., 40 μg/mL) will yield absorbed fluorescein concentration higher than that with lower fluorescein concentration in the fixed administration time, as well as flow rate in the upper channel and the bottom channel. In general, fluorescein concentration directly affects the absorption. Administration time directly affects the absorption. The administration at a longer time (e.g., 4 h) yields more fluorescein absorption than that with shorter administration time in the fixed fluorescein concentration, flow rate in the upper channel, and flow rate in the bottom channel. Overall, administration fluorescein concentration and time may be very positive for absorption, while the flow rate in the bottom channel may influence excretion.

### 3.5. Modeling PK Profiles of Anti-Cancer Drug Administration in Microfluidic System

**Figure 6:**
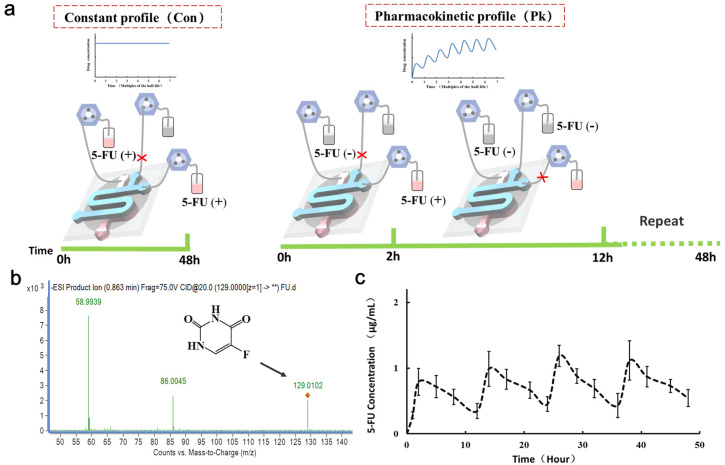
Modeling PK profiles of anti-cancer drug 5-FU administration in the microfluidic system. (**a**) Schematic drawing of two regimens of drug administration on chip. In the PK profile regimen (Pk group), 20 μg/mL 5-FU exposed cells for 2 h was then withdrawn for 10 h, over a period of 12 h. In the constant profile regimen (Con group), 5-FU (20 μg/mL) was continuously infused into the channel and treated cells for 48 h. (**b**) Detection of 5-FU peaks by mass spectrometry (MS) after treating cells with 5-FU for 48 h. (**c**) Characterization of concentration-time curve after treatment of cells with 20 μg/mL 5-FU for 2 days.

Drug administration regimens have a direct effect on how drug concentrations change over time, playing an important role in determining the pharmacological activity. Here, we present that microfluidic technology may be able to fill some of the unmet need in drug evaluation and PKs in vitro. We mimicked the absorption and clearance of the drug from the blood after oral by perfusing fresh medium in lower chip over time. Absorption plays a critical role in determining the activity and toxicity of various drugs. We applied a representative antineoplastic drug 5-FU, which exhibits cytotoxicity on the cancer cells, as a test drug for evaluation. As shown in Figure 6, we mimicked drug absorption and clearance profiles for human orally twice daily with 5-FU (20 μg/mL) treatment for 2 h under peristaltic flow and withdrawn for 10 h during a period of 12 h in PK profile regimen group. In constant profile group, cells were continuous treated with 20 μg/mL 5-FU for 48 h.

After the intestine cells cultured in the top chamber were exposed to 20 μg/mL 5-FU for 48 h, the conditioned medium from the chambers of target cells was collected to detect the drug absorbed 5-FU. To estimate the 5-FU concentration on intestine chip, we made standard curves at the same experimental condition. In order to compare the 5-FU absorbed from intestine cells, we detected the medium collected from the bottom channel using MS. The concentration-time curve obtained was shown in Figure 6c. The results demonstrated a dynamically changed concentration of the drug between 0 to 1 μg/mL in the PK profile group, which results in a different drug activity in target cells. In addition, the quantity of 5-FU shows dynamic profile of absorption and excretion in PK profile group, indicating the ability of the intestine chip to mimic the PK profile.

### 3.6. Assessment Inhibitory Effects of 5-FU on Target Cells

The different drug administration regimens can lead to significantly different pharmacological activity. A main reason is that drug concentration changes with different administration regimens [24]. This work proved that simply PK profiles could recreated in vitro in a multi-layered chip. While previous studies have analyzed FU activity on tumor cells in vitro, this is the first report of microfluidic chip modeling concentration-time curve.

**Figure 7:**
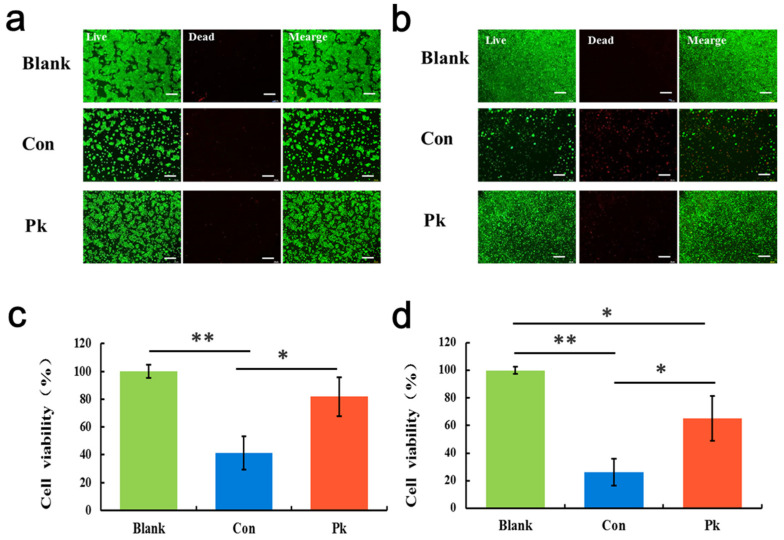
Effects of 5-FU on the viability of target cells on chip with different drug administration regimens. (**a**) HPAEpiC and (**b**) A549 cells were exposed to 5-FU (20 μg/mL) for 2 days under Blank, Con, and Pk groups, respectively. The cell viability was examined by the Live (green)/Dead (red) kit. Bars: 100 μm. Quantitative analysis of cell viability in (**c**) HPAEpiC and (**d**) A549 cells after treatment with 5-FU (20 μg/mL) for 2 days using CCK-8 kit. N = 3, * *p* < 0.05, ** *p* < 0.01.

Continuous exposure of the cells to different drug concentrations makes them particularly susceptible to different stimuli. We further performed two regimens of drug (5-FU) treatment on cells in this device. Briefly, the target cells within separated micro-chambers were exposed to 20 μg/mL 5-FU for 2 days and examined by Live/Dead kit staining and CCK-8 viability assay. 5-FU treatment is reported to be able to induce cells toxicity [33]. As shown in Figure 7, in the constant profile group, we found that the numbers of cells at the end of the 5-FU exposition was reduced when compared to the blank group. 5-FU has a relatively high on the viability of cells in PK profile group. The results indicated the 5-FU absorbed through intestine cells, leading to cytotoxicity on target cells, while the PK profile group had weakened toxicity with lower and dynamic drug concentration.

**Figure A1:**
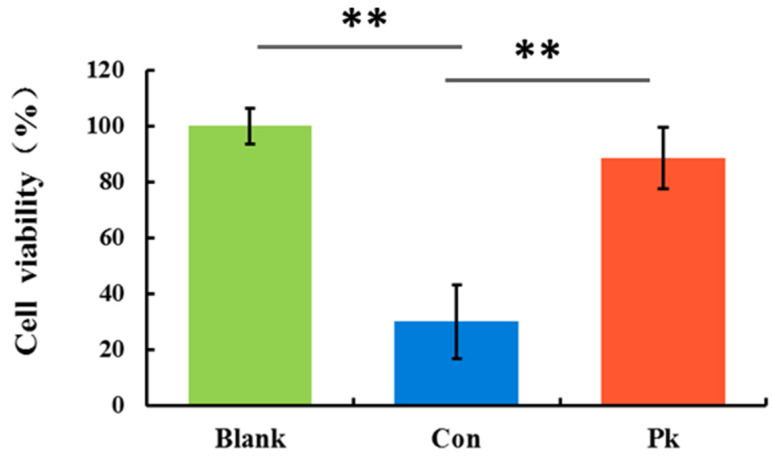
Effects of 5-FU on the viability of Caco-2 cells on chip with different drug administration regimens. Caco-2 cells were exposed to 5-FU (20 μg/mL) for 2 days under Blank, Con, and Pk groups, respectively. Quantitative analysis of cell viability in Caco-2 cells after treatment with 5-FU (20 μg/mL) for 2 days using CCK-8 kit. N = 3, ** *p* < 0.01.

The drug concentration in the PK profile group was dynamically changed from 0 to 1 μg/mL compared to the high concentration of 20 μg/mL of the constant profile group. Simultaneously, in PK profile group, we found that the cell viability of Caco-2 at the end of the 5-FU exposure maintain a high level shown in Figure A1. The cell viability of Caco-2 in constant profile group was low, which affects the function of barrier and absorption. It indicates that the PK profile administration regimen could make better use of the intestinal system for drug testing. Although due to technical limitations, the process of excretion is simply replaced by fluid within channel, the dynamic PK profiles of drug administration are more in line with the actual situation in vivo. It is clear that drug absorption processes is critical in determining its toxicity and pharmacological effect. Our results appeared encouraging for future development of functional organ model in microfluidic biochips. Taken together, these results showed the PK drug toxicity of 5-FU on target cells. The established approach enables the earlier assessment and toxicity prediction on target cells in a dynamic manner as existing in vivo. We illustrate the potential to fill in PK gaps between in vitro and in vivo using microfluidic system.

## 4. Conclusions

In summary, we developed a dynamic multi-layered microsystem containing intestine and targeted cells that allows mimicking of the in vivo-like drug concentration profile of anti-cancer drug 5-FU to targeted cells. The microfluidic system consists of compartmentalized chambers facilitating the co-culture of different cell types. Peristaltic flow through this system generates a low level of shear stress to improve intestinal barrier function and enhance cell layer integrity. The microfluidic system recapitulated both the absorption and excretion portions of drugs by integrating with peristaltic flow. We found the significant inhibition of 5-FU induced cytotoxicity on target cells at the PK profile group, primarily driven by the dynamic drug concentration profile affected by absorption. These results demonstrated the capability of this microfluidic system to mimic the PK profile of drug administration by control over flow rate, drug concentrations, and exposure time. This established microfluidic system may provide a valuable tool for validating dynamic dosing, preclinical drug testing, and predicting drug safety. This system can be further improved by integrating additional microfluidic elements, holding promising applications in drug screening and development.
